# Reasonable Timing of Radiotherapy for Stage IV Non-Small-Cell Lung Cancer During Targeted Therapy Based on Tumour Volume Change

**DOI:** 10.3389/fonc.2021.705303

**Published:** 2021-09-23

**Authors:** Qingsong Li, Na Liang, Xia Zhang, Yi Zhang, Weiwei Ouyang, Shengfa Su, Zhu Ma, Yinxiang Hu, Yichao Geng, Xiaxia Chen, Bing Lu

**Affiliations:** ^1^ Department of Oncology, Affiliated Hospital of Guizhou Medical University, Guiyang, China; ^2^ Department of Oncology, Guizhou Cancer Hospital, Guiyang, China; ^3^ Department of Oncology, Guiyang Guihang Hospital, Guiyang, China

**Keywords:** non-small-cell lung cancer, targeted therapy, tumour volume change, radiotherapy, reasonable timing

## Abstract

**Purpose:**

The aim of this study was to investigate the reasonable timing of radiotherapy for stage IV non-small-cell lung cancer (NSCLC) with EGFR-positive mutations during targeted therapy based on tumour volume change (TVC).

**Patients and Methods:**

Simulation Computed Tomography Scan (SCTS) measurements were taken to test TVC in patients with stage IV NSCLC during targeted therapy at intervals of 10 days. The SCTS measurement was terminated when the tumour volume shrinkage rate in the latter simulation compared with the previous simulation was ≤5% or when the time after treatment was 90 days. Then, primary tumour radiotherapy was performed. Related parameters of the radiotherapy plan were compared between the implementation and simulation plans.

**Results:**

Twenty-seven patients were enrolled in the analysis. After treatment, shrinkage of the primary tumour was observed in all patients, but the rate and speed were inconsistent. The average tumour volume decreased obviously within 40 days and was significantly different every 10 days (P ≤ 0.001). The average volume decreased slowly and tended to be stable (P>0.05) after 40 days. After the termination of SCTSs, 21 patients accepted primary tumour radiotherapy. No patients experienced grade 3+ acute radiation toxicity. The implementation radiotherapy plan was significantly better than that before treatment (all P<0.05) but not better than that on the 40th day after treatment (all P>0.05).

**Conclusions:**

To obtain a high radiation dose and control radiation toxicity, the 40th day after targeted therapy may be a reasonable time to start radiotherapy for stage IV NSCLC with EGFR-positive mutations.

**Clinical Trial Registration:**

https://www.clinicaltrials.gov/ct2/show/NCT03258671, identifier, NCT03258671.

## Background

First-line treatment of stage IV non-small-cell lung cancer (NSCLC) has evolved from chemotherapy alone to chemotherapy, targeted therapy, and immunotherapy (1), and targeted therapy for patients with positive mutations in driver *G*enes, such as human epidermal growth factor receptor (EGFR), anaplastic lymphoma kinase (ALK)/C-ros oncogene 1 receptor tyrosine kinase (ROS1) and T790M, has been shown to significantly prolong progression-free survival (PFS) (2–5). Higginson DS (6) et al. analysed stage III/IV NSCLC patients who received only systemic chemotherapy and found that the state of the primary tumour (large central tumour, pulmonary symptoms, and bronchial or vascular compression) was associated with poor OS. More importantly, recent studies have shown that targeted therapy, chemotherapy, and immunotherapy combined with three-dimensional radiotherapy of primary tumours and metastatic lesions can significantly improve overall survival (OS) (7–9) and significantly reduce the treatment failure rate of primary tumours from 80%-90% to less than 30% (10). A meta-analysis suggested that primary tumour radiotherapy, especially with radical doses, might further prolong survival (11). The local failure (12) was 82% for stage IV NSCLC treated with only EGFR-TKI. Previous studies (13, 14) showed that targeted therapy can increase the sensitivity of radiotherapy, and the combination therapy has the best inhibitory effect on cancer cell proliferation compared with radiotherapy alone or targeted therapy alone. OS benefits may be derived from the synergistic combination of radiotherapy and targeted therapy (15–18). However, the tumour burden of stage IV NSCLC is relatively large, with T_3_-_4_ accounting for 60%-70% and N_2-3_ accounting for 70%-80%, and the median volume of the primary tumour is approximately 134 cm^3^ (7, 19). The large tumour volume results in a low local control rate (LCR) due to the low radiation dose to reduce the rates of severe radiation toxicities and can also lead to an increase in radiation-induced toxicities due to an increased radiation dose. Therefore, we designed a prospective clinical trial to reduce the tumour to a certain size and maintain a relatively stable state by using EGFR-tyrosine kinase inhibitors (EGFR-TKIs), which have an objective response rate (ORR) of more than 70%, to realize the reasonable timing of radiotherapy to reduce normal tissue toxicity and increase the radiation dose, and to provide a reference for further randomized controlled studies on the reasonable timing of radiotherapy.

## Materials and Methods

### Patients, Study Design, and Treatment

The inclusion criteria were as follows: (1) pathologically confirmed, positive for sensitive driver *G*ene mutations, primary stage IV NSCLC (Union for International Cancer Control,UICC version 8), (2) no previous history of tumour treatment; (3) Karnofsky performance status (KPS)≥70; (4) aged from 18 to 80 years; (5) no contraindications to targeted therapy and radiotherapy; (6) signed informed consent; (7) clear consciousness when the metastatic sites were brain; (8) no influence on pulmonary function when the metastatic sites were lung; and (9) Normal bone marrow and organ function as defined below(absolute neutrophil count ≥ 1,500/mcl, Platelets ≥ 100000/mcl, Haemoglobin ≥ 9.0 g/Dl, Total bilirubin ≤ 2.0 x IULN(institution’s upper limit of normal), SGOT (serum glutamic-oxaloacetic transaminase)/SGPT (serum glutamic-pyruvic transaminase) ≤ 3.0 x IULN; if liver metastases, number ≤ 5.0, Serum creatinine ≤ 1.5 x IULN; LVEF (left ventricular ejection fraction) ≥ 50% performed no more than 4 weeks prior to enrolment; FEV1 (forced expiratory volume in the first second)>50%, mild-moderate pulmonary function dysfunction).

Tumour volume measurement process: (1) A Simulation Computed Tomography Scan (SCTS) was planned within 1 week before targeted therapy and every 10 days after the first day of treatment that patients underwent one simulation scan in sequence for a maximum of 90 days; (2) the SCTS within 1 week before targeted therapy was defined as C_0_; after the start of treatment, the SCTSs every 10 days were defined as C_10_-C_90_; the primary tumour volume before treatment (V_P_), volume of metastatic lymph nodes in the drainage area (V_N_) and gross tumour volume (GTV) were defined as V_P0_, V_N0_ and GTV_0_, respectively; and the volumes measured on the SCTSs were V_P10_-V_P90_, V_N10_-V_N90_ and GTV_10_-GTV_90_, respectively; (3) termination criteria for the SCTS were a tumour volume shrinkage (TVS) rate ≤5% in the latter simulation compared with the previous simulation or when the time after treatment was 90 days.

Delineation and calculation of tumour volume: Intensity-modulated radiotherapy (IMRT) was given *via* 6 MV X-ray. The patient was positioned in the supine position with thermoplastic film fixation, and the 5-mm-thick enhanced Computed Tomography (CT) scans were transferred to the Pinnacle3 planning system. V_P_ was outlined with a lung window (W: 1,600, L: -300), and V_N_ was outlined with a mediastinal window (W: 400, L: 800). Tumour volume was calculated, and the GTV compromised V_P_ and V_N_. The GTV was outlined on the last simulation CT image. The clinical target volume (CTV) was defined as the GTV plus a margin of 0.6 cm, and the planning target volume (PTV) was defined as the CTV plus another margin of 0.5 to 1.0 cm. The TVS rate of C_N_ was calculated as follows: TVS rate = (pre-treatment volume - simulation volume of C_N_)/pre-treatment volume × 100%.

Implementation radiotherapy plans and simulation plans: (1) IMRT was given *via* 6 MV X-ray. The implementation radiotherapy plans were created with the last simulation CT image. The radiotherapy dose was given to patients according to the tolerability of normal tissue and was maintained at ≤76 Gy. For all individual treatment plans, the percentage of the total lung volume receiving ≥20 Gy (V_20_) was maintained at ≤32% (≤25% in crizotinib-treated patients), V_5_ at ≤ 70%, mean lung dose (MLD) at ≤20 Gy, mean heart dose (MHD) at ≤26 Gy and maximum point dose to the spinal cord (SC-MPD) at ≤50 Gy. Radiotherapy plans were evaluated as 100% of the prescription dose line including 100% of the GTV and 95% of the prescription dose including 95% of the PTV or 90% of the prescription dose including 98% of the PTV. Patients received late-course accelerated hyperfractionated radiotherapy (LCAHRT) (20–23) to the primary tumour. The first course of radiotherapy was given in 1.8-Gy fractions, 5 days per week, to a total dose of 36-40 Gy/18-20 f. LCAHRT was then delivered in twice-daily fractions of 1.5 Gy each, separated by 6 to 8 hours per day, to a total dose of 21-30 Gy/14-20 f.

Simulation plans were created with the pre-treatment simulation (C_0_) and 40 days post-treatment (C_40_) simulation images. Implementation radiotherapy plans were adjusted according to the same dose or the same radiation toxicity control criteria for each patient, and the dose-volume histogram (DVH) was recorded.

### Drug Treatment

Gefitinib (250 mg, qd), erlotinib (150 mg, qd), icotinib (125 mg, tid) or crizotinib (250 mg, bid) was given according to the status of driver *G*ene-positive mutations. None of the patients received systemic chemotherapy.

### Radiotherapy to Metastatic Lesions

For oligometastatic NSCLC, all metastatic lesions were treated with radiotherapy. For non-oligometastatic NSCLC, radiotherapy to metastatic lesions was determined by clinical necessity, such as, brain metastasis, bone metastasis with cancer pain or risk of fracture.

Study endpoints and statistical methods: The primary endpoints were the change patterns of the V_P_, V_N_ and GTV before and during treatment, and the secondary endpoints were acute radiation pneumonitis (RP) (within 3 months after the end of radiotherapy), oesophagitis (RE) (NCICTC 3.0 criteria) and DVH parameters. Statistical analysis was performed using SPSS software (version 23.0). Measurement data are expressed as the mean ± standard deviation (SD) and were analysed with t-tests or Mann-Whitney U-tests. P<0.05 was considered a statistically significant difference.

## Results

### Patient Characteristics

**Figure 1:**
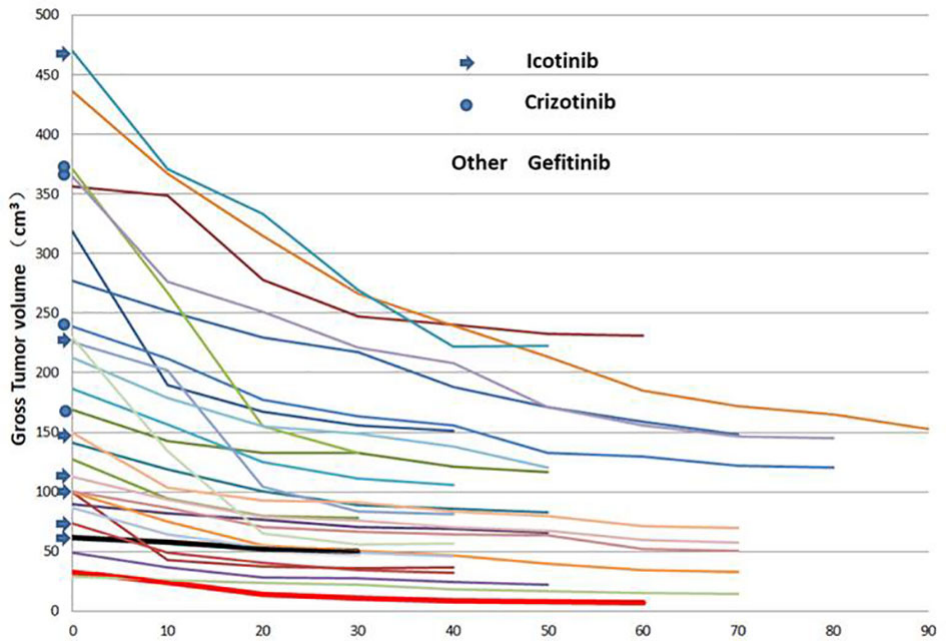
Changes in the primary tumour volume at different times after targeted therapy in 27 patients.

Thirty patients met the inclusion criteria, and 27 patients were eligible for analysis (refusal in 1 patient and SCTS not as planned in 2 patients). The ratio of males to females was 1.25, and the median patient age was 60 years. The most common site of metastatic disease at diagnosis was the bone, brain and lung (**Table 1**). The V_P0_, V_N0_ and GTV_0_ were 6.23~470.00, 0~362.97 and 28.86~470.00 cm^3^, respectively. The median and average GTV_0_ were 149.42 cm^3^ and 189.23 ± 127.03 cm^3^, respectively (the rest are shown in **Table 1**). Twenty-seven patients completed the SCTS and volumetric measurements according to the termination criteria. Twenty-three patients harboured EGFR-positive mutations: an exon 19 deletion mutation (19del) was observed in 14 patients, and an exon 21 deletion mutation (L858R) was observed in 9 patients. Four patients harboured an ALK rearrangement. Targeted therapy involved gefitinib in 16 patients, icotinib in 7 patients and crizotinib in 4 patients (**Figure 1**).

**Table 1:** Clinical characteristics of 27 patients.

Factor	No. (%)	Factor	No. (%)
Sex		T stage	
male	15 (56)	T_1_-T_2_	10 (37)
female	12 (44)	T_3_-T_4_	17 (63)
KPS		N stage	
70	1 (4)	N_0_-N_1_	17 (63)
80	15 (56)	N_2_-N_3_	10 (37)
90	11 (40)	M stage	
Age		M_1b_	20 (74)
40~64	21 (78)	M_1c_	7 (26)
65~75	6 (22)	Metastatic organ	
Smoking history		Bone	12 (44)
yes	9 (33)	Brain	9 (33)
no	18 (67)	Lung	5 (19)
Location		Other	5 (19)
Upper	12 (44)	Median number of metastatic lesions(Range)	
Middle-lower	15 (56)	All	1 (1-4)
Histology		Bone	1 (1-4)
Adenocarcinoma	26 (96)	Brain	1 (1-3)
NA	1 (4)	Lung	1 (1-2)
Type		Other	2 (1-2)
central	14 (52)		
peripheral	13 (48)		

### The Pattern of Tumour Volume Change

**Figure 2:**
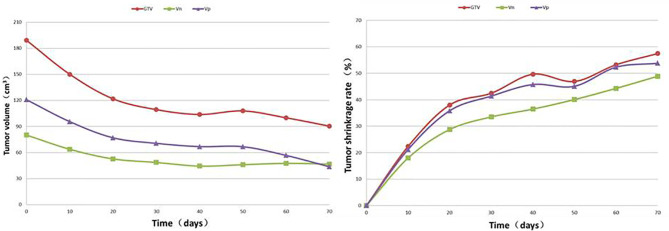
Regularity of the average value and shrinkage rate for the V_P,_ V_N_ and GTV at different times after targeted therapy in 27 patients.

SCTSs from C_0_ to C_30_ were performed on 27 patients, and C_40_, C_50_, C_60_, C_70_, C_80_ and C_90_ were performed on 24, 17, 11, 9, 3, and 1 patients, respectively. The GTV of all patients had different degrees of change from C_0_ to the last SCTS and showed a trend of gradual shrinkage, in which the largest volume shrinkage rate was 78.1% (gefitinib, thick red solid line) and the smallest was 18.8% (icotinib, thick black solid line) (**Figure 1**). According to the graph of mean GTV, V_P_, and V_N_ (C_0-70_) values, tumour volume decreased gradually and significantly within the 40th day after treatment and then tended to stabilize (3 patients in C_80_ and 1 patient in C_90,_ not analysed). The mean tumour volume continued to shrink or tended to stabilize after slightly increasing at 50 days (**Figure 2**).

### GTV Changes in Two Adjacent SCTSs

GTV changes in two adjacent SCTSs showed that the tumour volume shrinkage rate was inconsistent before the C_40_ SCTS every 10-day interval, and the tumour volume shrinkage rate was <5% on the C_40_ and C_50_ SCTSs and every 10-day interval thereafter (**Table 2**).

**Table 2:** Comparison of the gross tumour volume (cm^3^) every 10 days after targeted therapy in 27 patients.

Factor	Gross tumour volume (cm^3^)		*P* value
C_0_vsC_10_	189.23 ± 127.03	150.15 ± 105.64	<0.001
C_10_vsC_20_	150.15 ± 105.64	121.92 ± 90.53	<0.001
C_20_vsC_30_	121.92 ± 90.53	109.50 ± 77.64	<0.001
C_30_vsC_40_	109.50 ± 77.64	103.92 ± 72.74	0.001
C_40_vsC_50_	103.92 ± 72.74	107.44 ± 73.13	0.969
C_50_vsC_60_	107.44 ± 73.13	100.08 ± 75.28	0.677
C_60_vsC_70_	100.08 ± 75.28	90.48 ± 57.30	0.710

### Volume and Shrinkage Rates of Tumours at Different Times After Treatment

The change patterns in V_P_ and V_N_ were similar to that of the GTV after treatment, with the most significant shrinkage rate in the first 10 days (C_10_). The shrinkage rates of the GTV_10-40_ were 22.21%, 14.64%, 5.54% and 4.37%, respectively. In every interval from C_40_ to C_90_, 3, 7, 6, 2, 6 and 2 patients met the termination criteria due to having a shrinkage rate (adjacent comparison) <5%. The average shrinkage rate from C_40_ to C_70_ was 2.67%. Only 1 patient continued to have a >5% shrinkage rate at C_90_ (**Table 3**).

**Table 3:** Changes in the gross tumour volume (GTV), primary tumour volume (V_P_), and metastatic lymph nodes in drainage areas (V_N_) at different times after targeted therapy in 29 patients (mean ± SD).

Item	No.	V_P_		V_N_		GTV	
		volume (cm^3^)	shrinkage (%)	volume (cm^3^)	shrinkage (%)	volume (cm^3^)	shrinkage (%)
C_0_	27	120.92 ± 122.54		73.79 ± 90.11		189.23 ± 127.03	0
C_10_	27	95.62 ± 100.96	22.36 ± 18.30	58.71 ± 68.62	17.23 ± 13.84	150.15 ± 105.64	21.21 ± 12.35
C_20_	27	77.06 ± 85.37	38.04 ± 24.58	48.52 ± 53.36	28.11 ± 15.94	121.92 ± 90.53	35.85 ± 15.29
C_30_	27	68.03 ± 72.44	42.48 ± 20.58	44.77 ± 47.88	32.99 ± 15.41	109.50 ± 77.64	41.39 ± 15.35
C_40_	24	66.87 ± 66.05	49.63 ± 16.28	40.42 ± 45.47	36.27 ± 13.13	103.92 ± 72.74	45.76 ± 13.62
C_50_	17	66.81 ± 70.10	46.91 ± 13.72	43.17 ± 41.74	39.53 ± 12.07	107.44 ± 73.13	45.10 ± 11.94
C_60_	11	56.79 ± 63.02	53.23 ± 14.85	47.61 ± 44.89	44.02 ± 11.88	100.08 ± 75.28	52.31 ± 11.85
C_70_	9	43.71 ± 44.07	57.44 ± 11.45	46.77 ± 45.61	48.20 ± 9.80	90.48 ± 57.30	53.76 ± 7.02

### Acute Radiotherapy Toxicity

Twenty-one patients (6 of whom refused radiotherapy after the termination of simulation) were treated with primary tumour radiotherapy according to the last CT simulation image and were followed up until 90 days after the end of radiotherapy. There were 5 (23.8%), 2 (9.5%), 1 (4.8%) and 5 (23.8%), 3 (14.3%), 0 (0%) cases of grade I, II and III acute RP and RE, respectively.

### Comparison of DVH Parameters Between the Implementation Plan and the Corresponding Simulation Plan

The comparison between the implementation plan and the corresponding simulation plan with the same primary tumour dose revealed the following. The lung V_20_, MLD, and MHD of the C_0_ plan were significantly higher than those of the implementation plan and the C_40_ plan. The lung V_5_, SC-MPD and oesophageal V_50_ also tended to increase. The C_40_ plan was similar to the implementation plan (**Table 4**). The comparison between the implementation plan and the simulation plan with the same radiation damage control criteria revealed the following. The C_40_ plan increased the radiotherapy dose from 63 ± 7 Gy at C_0_ to 66 ± 7 Gy (P<0.001), and the implementation plan increased the radiotherapy dose to 68 ± 7 Gy (P<0.001). The radiotherapy dose of the C_40_ plan was similar to that of the implementation plan (P=0.110).

**Table 4:** Comparison of dose-volume histogram parameters in the pre-treatment localization (C_0_) and 40 days post-treatment (C_40_) simulation plans and implementation plans in 21 patients (mean and range).

Item	C_0_ plan	C_40_ plan	Implementation plan	P_1_	P_2_	P_3_
Lung V_5_ (%)	0.65 (0.60~0.72)	0.62 (0.54~0.67)	0.61 (0.55~0.67)	0.066	0.001	0.301
Lung V_20_ (%)	0.31 (0.27~0.36)	0.28 (0.24~0.32)	0.27 (0.22~0.32)	0.002	<0.001	0.149
Oesophagus V_50_ (%)	0.35 (0.21~0.50)	0.33 (0.21~0.47)	0.30 (0.15~0.47)	0.382	0.088	0.284
MHD (Gy)	25.42 (17.59~30.23)	23.66 (15.29~30.36)	21.70 (15.21~26.59)	0.040	0.001	0.090
SC-MPD (Gy)	46.57 (41.04~51.75)	44.62 (39.59~50.69)	44.42 (39.60~51.38)	0.083	0.063	0.899
MLD (Gy)	19.18 (15.80~22.99)	17.40 (14.00~21.55)	16.76 (12.44~19.29)	0.027	0.001	0.494

P_1_, C_0_ plan vs C_40_ plan; P_2_, C_0_ plan vs implementation plan; P_3_, C_40_ plan vs implementation plan.

## Discussion

The median survival time (MST) of patients with stage IV NSCLC who received three-dimensional radiotherapy to the primary tumour combined with chemotherapy was prolonged to 16 months, and radiotherapy may play a very important role in prolonging OS based on the benefits of systemic therapy (21). Stereotactic ablative radiotherapy and stereotactic body radiotherapy to the primary tumour or metastases combined with EGFR-TKIs or first-line chemotherapy (for patients without EGFR mutations) significantly prolonged PFS and OS in patients with oligometastatic NSCLC (16, 24–28). Increasing the radiotherapy dose to the primary tumour was strongly associated with improved OS, and a radical radiation dose may be more beneficial for OS, especially in patients with oligometastases (10, 21, 24, 26). Radiotherapy has become an important treatment for prolonging OS by reducing the failure rate of the primary tumour in patients with stage IV NSCLC (21). However, it is well known that the radical radiation dose can improve the LCR. However, the volume of the irradiated target area is an important factor that affects an increase in the tumour dose and controls radiation injury to normal tissues (27). The large irradiated volume leads to the phenomenon that radiation injury is aggravated by high doses to improve the LCR, or the LCR is reduced by low doses for fear of radiation injury. Therefore, reducing the volume of the irradiated tumour is the key to both increasing the dose and LCR and decreasing the incidence of radiation injury. However, the primary tumour is large in volume and scattered, and patients mainly have T_3-4_/N_2-3_ disease according to research data (11, 21, 24). In some patients, when radiotherapy and EGFR-TKIs are started simultaneously, the purposes of both increasing the dose to the primary tumour and protecting normal tissues from radiation injury cannot be achieved because of the primary tumour volume. Therefore, this study was designed to take advantage of the ORR of EGFR-TKI treatment (>70%), disease control rate (>90%), and PFS (9-11 months) (29–31) based on the dosimetric property that a ≥15% shrinkage rate in the primary tumour volume can significantly reduce the low-dose volume to the whole lung and reduce radiation injury (27). Patients underwent SCTSs before EGFR-TKI treatment and every 10 days after treatment. The SCTS measurement was terminated, and then primary tumour radiotherapy began when the TVS in the latter simulation compared with the previous simulation was ≤5% or when the time after treatment was 90 days. The aim was to investigate the timing of administering radiotherapy to the primary tumour to both increase the dose and LCR and reduce the probability of radiation injury.

This study showed that although each patient had positive mutations in driver *G*enes, the rate and degree of tumour volume shrinkage after EGFR-TKI treatment were not consistent. Until the last SCTS, the maximum and minimum shrinkage rates were 78.1% and 18.8%, respectively. The most significant change in the average volume was within 40 days after the start of treatment. Thereafter, the average volume shrinkage rate slowed and was relatively stable at every 10-day interval. The total and average shrinkage rates from C_40_ to C_70_ were 8% and 2.67%, respectively. On day 50, the shrinkage rate increased slightly (by 3%) and continued to decrease thereafter. The regularity of TVC after EGFR-TKI treatment is that the volume shrinkage gradually slows the volume continues to shrink after increasing in some cases, and tumour shrinkage varies due to the different sensitivities of EGFR-TKI treatment in different patients (32). Therefore, it may be most beneficial to start radiotherapy at the time when the tumour volume continues to shrink to a low level after treatment and stabilizes without waiting until the disease progresses. In this study, the primary tumour volume was measured and compared separately at each 10-day interval. The results showed that the tumour volume shrinkage rates were significant and different within 40 days after the start of treatment. The tumour volumes from days C_40_ to C_70_ were similar and slow, and the tumour volume increased slightly on day 50 in 1 patient, which suggests that the speed of tumour volume shrinkage is different in each individual. For patients who receive EGFR-TKI treatment, a certain regularity of tumour volume shrinkage may be deduced, or 60 days may be the time to carry out radiotherapy by means of mathematical modelling (33), but an individualized analysis was not performed, and the actual pattern of TVC was not examined. Therefore, the current study shows that TVS was significant within 40 days after EGFR-TKI treatment and entered the stable phase after 40 days in most patients. The 40th day after EGFR-TKI treatment may be a reasonable time to administer radiotherapy to reach the goals of controlling tumours and reducing injury.

The simulated radiotherapy plan and its parameters represent the dose likely to control the primary tumour and the threshold to protect normal tissues from radiation injury (34, 35), while the implementation radiotherapy plan and its parameters validate and summarize the efficacy for each individual tumour and the probability of radiation injury for normal tissues after a given dose of radiotherapy (36). Grade 2 and 3 acute RP and RE were observed in only a small number of patients treated with radiotherapy after the termination of SCTSs in this study, which suggests that the safety and efficacy of radiotherapy are acceptable under the premise of injury control criteria. The simulation radiotherapy plans for the primary tumour at C_0_ and C_40_ were designed at the same dose as the implementation plans of the corresponding patients. The DVH parameters of the 3 plans were compared. The results showed that compared to the C_0_ simulation plan, the implementation plan and the C_40_ plan significantly reduced the lung V_20_, MLD, and MHD. There was a trend of a significant reduction in the lung V5 and SC-MPD. The reduction in the lung V_20_ may significantly reduce the occurrence of RP (28). There was a trend of a significant reduction in the oesophageal DVH of the implementation plans that may reduce the incidence of RE. Under the premise of the same control criteria of radiation injury, the radiation doses were compared among the implementation plan and the C_0_ and C_40_ simulation plans. The results showed that the implementation plan and C_40_ simulation plan could significantly increase the tumour dose compared with the C_0_ simulation plan and achieved a radical dose of more than 60 Gy, which not only improved the LCR but also did not increase radiation injury (34–37). The implementation plan was similar to the C_40_ simulation plan in both the tumour dose and DVH parameter regarding radiation injury protection. Therefore, it was further validated that it is a reasonable time to start primary tumour radiotherapy at 40 days after EGFR-TKI treatment in patients with EGFR-positive mutations.

In summary, the tumour volume shrinkage rate after EGFR-TKI treatment in patients with stage IV NSCLC with driver *G*ene-positive mutations gradually slowed over time and varied in each individual. The shrinkage rate was significant within 40 days after treatment and then entered the stable stage, and it may be the best time to start radiotherapy after 40 days of the initial treatment.

## Data Availability Statement

The raw data supporting the conclusions of this article will be made available by the authors, without undue reservation.

## Ethics Statement

The studies involving human participants were reviewed and approved by the Ethics Committee of Guizhou Cancer Hospital, GuiYang, China. The patients/participants provided their written informed consent to participate in this study.

## Author Contributions

QL, NL, XZ, YZ, WO, SS, ZM, YH, YG, and XC collected the data. BL conceived the study and participated in its design and coordination. BL and QL performed the statistical analysis and drafted the manuscript. All authors contributed to the article and approved the submitted version.

## Funding

Guizhou Science and Technology Plan Support Project [Qiankehe support (2019) 2795].

## Conflict of Interest

The authors declare that the research was conducted in the absence of any commercial or financial relationships that could be construed as a potential conflict of interest.

## Publisher’s Note

All claims expressed in this article are solely those of the authors and do not necessarily represent those of their affiliated organizations, or those of the publisher, the editors and the reviewers. Any product that may be evaluated in this article, or claim that may be made by its manufacturer, is not guaranteed or endorsed by the publisher.
